# CYP3A-status is associated with blood concentration and dose-requirement of tacrolimus in heart transplant recipients

**DOI:** 10.1038/s41598-021-00942-y

**Published:** 2021-11-01

**Authors:** Máté Déri, Zsófia Szakál-Tóth, Ferenc Fekete, Katalin Mangó, Evelyn Incze, Annamária Minus, Béla Merkely, Balázs Sax, Katalin Monostory

**Affiliations:** 1 Institute of Enzymology, Research Centre for Natural Sciences, Magyar tudósok 2, Budapest, 1117 Hungary; 2 Heart and Vascular Center, Semmelweis University, Városmajor 68, Budapest, 1122 Hungary

**Keywords:** Clinical genetics, Gene expression, Molecular biology, Molecular medicine

## Abstract

High inter-individual variability in tacrolimus clearance is attributed to genetic polymorphisms of CYP3A enzymes. However, due to CYP3A phenoconversion induced by non-genetic factors, continuous changes in tacrolimus-metabolizing capacity entail frequent dose-refinement for optimal immunosuppression. In heart transplant recipients, the contribution of patients’ CYP3A-status (*CYP3A5* genotype and CYP3A4 expression) to tacrolimus blood concentration and dose-requirement was evaluated in the early and late post-operative period. In low CYP3A4 expressers carrying CYP3A5*3/*3, the dose-corrected tacrolimus level was significantly higher than in normal CYP3A4 expressers or in those with CYP3A5*1. Modification of the initial tacrolimus dose was required for all patients: dose reduction by 20% for low CYP3A4 expressers, a 40% increase for normal expressers and a 2.4-fold increase for CYP3A5*1 carriers. The perioperative high-dose corticosteroid therapy was assumed to ameliorate the low initial tacrolimus-metabolizing capacity during the first month. The fluctuation of CYP3A4 expression and tacrolimus blood concentration (C_0_/D) was found to be associated with tapering and cessation of corticosteroid in CYP3A5 non-expressers, but not in those carrying CYP3A5*1. Although monitoring of tacrolimus blood concentration cannot be omitted, assaying recipients’ CYP3A-status can guide optimization of the initial tacrolimus dose, and can facilitate personalized tacrolimus therapy during steroid withdrawal in the late post-operative period.

## Introduction

Heart transplantation is an effective treatment option for patients with end-stage heart dysfunction refractory to maximal medical management; however, due to the gap between waiting list and eligible organs, it is available only for a limited number of patients^1,2^. Improvement in surgical techniques, intensive care and immunosuppression has led to increasing recipient survival^3,4^. According to the International Society for Heart and Lung Transplantation database, the one-year survival among heart transplant recipients was over 85% (https://ishlt.org/research-data/registries/ttx-registry/ttx-quarterly-data-report, access: 22.07.2021), and further efforts are made to minimize the risk of graft dysfunction after transplantation^3,5^. However, complications in the postoperative period, such as acute rejection, infection and renal insufficiency, are still a challenge for clinicians^6–8^. One of the causes, associated with these conditions is recipients’ variability in immunosuppressant pharmacokinetics that also contributes to post-transplant outcome.

Recipients' drug therapy primarily focuses on immunosuppression and control of allograft rejection as well as on prevention of infections and avoidance of adverse effects of immunosuppressants^8,9^. Intense initial induction therapy with polyclonal or monoclonal antibodies is applied in about half of heart transplantations, in the remaining cases, lifelong maintenance immunosuppressive therapy follows immediately after surgery^5,10^. The conventional maintenance immunosuppression generally consists of corticosteroids (prednisone, prednisolone or methylprednisolone), calcineurin inhibitors (cyclosporine A or tacrolimus) and antimetabolite drugs (azathioprine, mycophenolate mofetil or mycophenolic acid)^4,11^. Calcineurin inhibitor therapy is the cornerstone of maintenance immunosuppressive regimens with tacrolimus, the most frequently used agent^4,12^. Because of the narrow therapeutic range and the large pharmacokinetic variability among individuals, continuous monitoring of tacrolimus blood concentrations is essential for optimal therapeutic efficacy^12,13^. The non-balanced sub-optimal therapy leads to allograft rejection episodes, whereas supra-optimal therapy can result in nephrotoxicity, neurotoxicity or increased susceptibility to infections. Therefore, any factor that can modulate the blood concentrations of immunosuppressants, particularly of tacrolimus influences the outcome of transplantation^8,14,15^.

Tacrolimus is a substrate of ABCB1 transporter (ATP-binding cassette family B1) and CYP3A enzymes (cytochrome P450 3A)^16,17^; therefore, inter-individual variations in tacrolimus pharmacokinetics have been supposed to be associated with genetic variability of ABCB1 efflux transporter and drug metabolizing CYP3A enzymes^18,19^. Plenty of clinical studies investigated the impact of *ABCB1* functional polymorphisms on tacrolimus exposure in solid organ transplantation; however, clear association between *ABCB1* variants and tacrolimus bioavailability is debated^12,20,21^ Hepatic and intestinal tacrolimus-metabolizing capacity rather than *ABCB1* polymorphisms appear to influence tacrolimus bioavailability. Genetic polymorphisms of CYP3A enzymes (CYP3A5, CYP3A4), the key catalysts of tacrolimus metabolism, have been proposed to significantly contribute to inter-patient variability in tacrolimus clearance^12,19,21,22^. The single nucleotide polymorphism in CYP3A5*3 allele (6986A > G, rs776746), most common in Caucasian populations, results in truncated mRNA and absence of functional CYP3A5 enzyme^23,24^. The Clinical Pharmacogenetics Implementation Consortium has recommended *CYP3A5* genotype-guided tacrolimus dosing in order to rapidly achieve optimal blood concentration after initiation of tacrolimus therapy^25^. Additional genetic variants of *CYP3A4*, such as CYP3A4*1B and CYP3A4*22, have also been suggested to be taken into account during prediction of tacrolimus-metabolizing capacity^12,26,27^. CYP3A4*1B (-392A > G, rs2740574) appears to contribute to increased transcription of *CYP3A4* gene, and a close link between the wild-type CYP3A5*1 and CYP3A4*1B alleles has been reported which makes the relative contribution to tacrolimus metabolism indistinguishable^28,29^. CYP3A4*22 (15389C > T, rs35599367) has been proposed to be associated with low CYP3A4 expression and reduced activity^30^. However, the substantial inter-individual or even the intra-individual variability is hardly attributed merely to the genetic polymorphisms of *CYP3A*s. The *CYP3A* genotype determines the potential for the expression of functional or non-functional CYP3A enzymes, whereas non-genetic factors, such as co-medication with CYP3A-inducer drugs (e.g. glucocorticoids, rifampicin), can result in phenoconversion that significantly modulates CYP3A expression and tacrolimus clearance^31,32^. Patients’ calcineurin inhibitor metabolizing capacity can be characterized by the CYP3A-status (*CYP3A5* genotype and CYP3A4 expression). *CYP3A5* genotyping identifies the genetically determined CYP3A5 expressers or non-expressers, and CYP3A4 expression in leukocytes can estimate reduced or increased hepatic CYP3A4 activities. We have previously demonstrated a strong correlation between CYP3A4 mRNA expression in leukocytes and hepatic CYP3A4 activities^33^. It means that peripheral leukocytes are appropriate biological samples for providing information about CYP3A4 activities of the liver. In liver transplant recipients, we have successfully applied this complex diagnostic system (CYPtest™) and clearly demonstrated that CYP3A4 expression rates of liver donors combined with *CYP3A5* genotypes influenced blood concentrations of calcineurin inhibitors (tacrolimus, ciclosporin) in recipients^34^. The recipients with liver grafts from low or high CYP3A4 expressers or with grafts carrying CYP3A5*1 required substantial modification of the initial doses. Furthermore, CYP3A-status guided tacrolimus therapy significantly reduced the risk of misdosing induced acute rejection and nephrotoxicity^35^.

The clinical protocols of immunosuppressive regimens for heart transplant recipients are basically defined by the transplantation centre^8,36^; however, due to continuous changes of patients’ tacrolimus-metabolizing capacity, frequent refinement of dosing is required for balanced and optimal immunosuppression. The initial high-dose corticosteroid therapy is expected to increase CYP3A expression and the rate of tacrolimus metabolism that are abated during tapering and withdrawal of corticosteroids. The major aim of the present study was to investigate the influence of heart transplant patients’ CYP3A-status (*CYP3A5* genotype and CYP3A4 expression) on tacrolimus blood concentration and dose-requirement early after transplantation. Our further goal was to undertake a systematic evaluation of recipients’ CYP3A-status and tacrolimus-metabolizing capacity in the post-transplantation period (0–15 months) and to identify corticosteroid co-medication induced alterations that modified tacrolimus pharmacokinetics.

## Materials and methods

### Patients and study design

Adult, heart transplant patients (N = 232) at the Heart and Vascular Centre, Semmelweis University (Budapest, Hungary) were enrolled in the study. CYPtesting of the recipients and the study protocol were approved by the Committee of Science and Research Ethics, Medical Research Council (2112-2/2017/EKU, 32,911-2/2019/EKU), and the study was performed in accordance with the relevant guidelines and regulations (Act CLIV of 1997 on Health, decree 23/2002 of the Minister of Health of Hungary and the declaration of Helsinki). Each recipient gave the informed consent to participate in the study. Post-transplant CYP3A-status was determined in 232 recipients, whereas 163 patients were involved in the evaluation of CYP3A-status and post-operative tacrolimus blood concentration or dose-requirement. Of the 232 patients, 14 patients died within the first two weeks after surgery, while 55 patients were treated with strong CYP3A inhibitors (e.g. fluconazole, itraconazole) or were transferred to another healthcare institution shortly after transplantation; therefore, these patients (N = 69) were excluded from the analysis. CYP3A4 expression and tacrolimus blood concentrations were followed in 78 patients in the first 15 months after transplantation. The patients' demographic and clinical data (Table 1) as well as the details of tacrolimus therapy (dosage and pre-dose blood concentrations) were recorded. The post-transplant drug therapy was applied according to the conventional clinical protocol which included immunosuppressants and prophylactic medications, such as antibiotics (sulfamethoxazole-trimethoprim, ciprofloxacin, meropenem) and antiviral drugs (ganciclovir, valganciclovir), antihypertensive agents (lercanidipine, ramipril, perindopril), cholesterol-lowering agent (rosuvastatin), acid-reducing drugs (famotidine, pantoprazole) and if necessary analgesics (paracetamol, ibuprofen). In the late post-operative period, most of these drugs were withdrawn, whereas tacrolimus was applied as a life-long medication.

**Table 1:** Demographic data of the heart transplant patients.

Demographic data	CYP3A testing	CYP3A-status – tacrolimus therapy association
Number of patients	232	163
Age at the time of transplantation (year)^a^	53.1 (19.5; 68.7)	52.6 (19; 68.7)
Gender (male/female)	175/57	128/35
Bodyweight at the time of transplantation (kg)^a^	78 (47; 120)	80 (47; 120)
**Primary disease**		
Non-ischaemic DCM	101	68
Ischaemic CM	78	57
Congenital heart disease	17	11
Hypertrophic CM	9	8
Restrictive CM	8	5
ARVD	3	2
Idiopathic DCM	3	2
Other	13	10

ARVD, arrhythmogenic right ventricular dysplasia; CM, cardiomyopathy; DCM, dilated CM.

^a^Median (min; max).

### Immunosuppressive protocol and drug monitoring

The induction immunosuppressive therapy with anti-thymocyte globulin, high-dose corticosteroid (methylprednisolone) and mycophenolate mofetil started immediately after transplantation and lasted up to 4 days. The maintenance regimens consisted of tacrolimus, corticosteroid and mycophenolate mofetil. The initial corticosteroid dose of 250 mg was administered at the time of operation, and the subsequent doses were gradually tapering (125-16-12 mg/day) to a maintenance daily dose of 8 mg by the end of the 3rd week and thereafter. Corticosteroid dose was tapered to a median of 2 mg/day by the end of the first year and was completely withdrawn by the 15th month. Mycophenolate mofetil was applied at the daily dose of 3 g at the early post-operative period, and was tapered to the daily dose of 2 g when tacrolimus reached the therapeutic range (> 10 ng/ml). Tacrolimus therapy was started 5 days after heart transplantation and was administered twice a day. The daily dose was defined as the sum of the morning dose, given after the blood sampling for trough blood concentration (C_0_) measurement and the evening dose administered after 12 h later to the morning dose. The initial tacrolimus dose was adjusted to the recipients’ bodyweight (0.1 mg/kg) and thereafter controlled by the pre-dose blood concentrations according to the standard clinical protocol. The oral tacrolimus dosage was adjusted to a target therapeutic window in the range of 10–15 ng/ml in the first 6 months, of 8–12 ng/ml in the period of 6–12 months and of 5–10 ng/ml after 12 months.

Therapeutic drug monitoring of tacrolimus was performed routinely (every day in the first week of tacrolimus initiation, every second day from the second week and at least every month from the first month), and the dose was modified if the exposure was out of the target range of tacrolimus blood concentration. The 12 h post-dose trough concentrations of tacrolimus (C_0_) were determined in whole blood taken at around 6.00 am before the morning dose was administered. Pre-dose concentrations were calculated by dividing the C_0_ by the corresponding 24 h dose on a mg/kg bodyweight basis (C_0_/D). The blood concentrations were measured using enzyme immunoassay techniques for tacrolimus (TACR Flex Dimension Dade Behring Inc., Newark, DE) in which free and tacrolimus-bound antibody conjugates were separated using magnetic particles. Tacrolimus-bound antibody conjugates were detected by the β-galactosidase reaction hydrolysing chlorophenol red β-d-galactopyranoside. The assay range for tacrolimus was 1.1–33.6 ng/ml, the intra- and inter-day variability for the quantification was less than 10%. According to the assay description, cross-reactivity with some of the tacrolimus metabolites was detected, and in transplant patients, tacrolimus concentrations approximately 13% higher may be measured by TACR Flex Dimension than by LC–MS/MS (liquid chromatography coupled with tandem mass spectrometry) (https://www.accessdata.fda.gov/cdrh_docs/reviews/K060502.pdf, access: 04.10.2021).

### CYP3A-status of the heart transplant recipients

The CYP3A-status was assayed in peripheral blood of 232 heart transplant recipients. Blood sampling for *CYP3A* genotyping and for measurement of CYP3A4 expression was performed on the second postoperative day. For those who were involved in tacrolimus follow-up study, CYP3A4 mRNA expression was measured at 1, 3, 6, 12 and 15 months after transplantation. Leukocytes and genomic DNA were isolated from the peripheral blood samples according to the methods described by Temesvári et al.^33^. *CYP3A5* and *CYP3A4* genotyping were carried out by hydrolysis single nucleotide polymorphism (SNP) analysis for CYP3A5*3, CYP3A4*1B and CYP3A4*22 using TaqMan probes (Eurofins Genomics Germany GmbH, Ebersberg, Germany). The genotypes were distinguished by post-PCR (polymerase chain reaction) allelic discrimination plotting the relative fluorescence values for wild-type and mutant alleles^34^. For CYP3A4 expression, total RNA was extracted from leukocytes, and RNA (5 μg) was reverse transcribed into single-stranded cDNA using the Maxima First Strand cDNA Synthesis Kit (Thermo Scientific, Waltham, MA, USA). Real-time PCR with human cDNA was carried out by using Kapa Probe Fast qPCR Master Kit™ (Merck KGaA, Darmstadt, Germany) and TaqMan probe and primers specific for CYP3A4 (Eurofins Genomics Germany GmbH). The quantity of CYP3A4 mRNA relative to that of the housekeeping gene glyceraldehyde 3-phosphate dehydrogenase (GAPDH) was determined. Three categories of CYP3A4 expression were applied to describe low, normal and high expressers. The cut-off values for the CYP3A4 mRNA levels in leukocytes have been previously established on the basis of the cut-off values for the hepatic CYP3A4 activities (nifedipine oxidation or midazolam 1′- and 4-hydroxylation)^33^. Low expressers displayed a CYP3A4/GAPDH ratio less than 10^–6^, normal expressers a ratio between 10^–6^ and 10^–4^, whereas in high expressers the ratio was higher than 10^–4^. Primer and TaqMan probe sequences for genotyping and mRNA expression measurement are in the supplementary table (Supplementary Table S1).

### Data analysis

Linkage disequilibrium between *CYP3A5* and *CYP3A4* SNPs (rs776746 and rs2740574) was calculated using Haploview (v4.2; Broad Institute, Cambridge, MA)^37^. The recipients were categorized by their CYP3A-status. The patients carrying at least one CYP3A5*1 allele were considered to be CYP3A5 expressers, while recipients with the CYP3A5*3/*3 genotype were CYP3A5 non-expressers. The CYP3A5 non-expresser recipients were subdivided into three CYP3A4 expresser groups by their CYP3A4 mRNA levels. The blood concentration values of tacrolimus were normalized by the dose and the bodyweight of transplant recipients, and expressed as (ng/ml)/(mg dose/kg bodyweight). The data of normalized blood concentrations (C_0_/D) and dose-requirements for the optimal therapeutic level in the recipient groups with various CYP3A-statuses were expressed as the median and mean ± standard deviation (SD). It should be noted that median values generally did not differ much (mostly by 7–10%) from the mean values. Statistical analysis was carried out using GraphPad InStat (v3.05; Graph-Pad Software, San Diego, CA). Between group differences or variations in CYP3A4 mRNA expression and tacrolimus concentration at various time-points after transplantation were calculated by using Kruskal–Wallis analysis of variance followed by Dunn’s multiple comparisons test. A P value of < 0.05 was considered to be statistically significant.

## Results

### CYP3A-status of heart transplant recipients

The loss-of-function CYP3A5*3 allele was identified in heart transplant patients, whereas the wild-type CYP3A5*1 was assigned in the absence of CYP3A5*3. Of 232 heart transplant patients, 230 carried one or two CYP3A5*3 alleles (34 patients with CYP3A5*1/*3 and 196 with CYP3A5*3/*3 genotype) (Table 2), displaying allele frequency (91.8%) similar to that in Caucasian white populations (88–97%)^38^. Two polymorphic *CYP3A4* alleles (CYP3A4*1B, CYP3A4*22) that are supposed to influence CYP3A4 expression, were also identified. The majority of heart transplant recipients (more than 85%) carried CYP3A4*1/*1, whereas 13.4% were heterozygous (17 patients carried CYP3A4*1/*1B and 14 displayed CYP3A4*1/*22 genotype). One patient was homozygous for CYP3A4*1B; however, CYP3A4*22/*22 genotype was not detected in the 232 patients. The genetic linkage in the *CYP3A* locus is well-characterized^39^, and single nucleotide polymorphisms in CYP3A4*1B and CYP3A5*1 (rs2740574 and rs776746) were found to be in significant linkage in the 232 heart transplant patients (D’ 0.758; LOD 9.42). CYP3A4*22 or CYP3A4*1B alleles, assumed to result in reduced and increased expression of CYP3A4, respectively^40^, were identified in 32 patients. However, no association was found between various *CYP3A4* genotype groups and CYP3A4 expression probably due to the low frequencies of the polymorphic *CYP3A4* alleles (CYP3A4*1B: 4.09%; CYP3A4*22: 3.02%) in the heart transplant recipients and/or to non-genetic factors that masked the effect of genotype on CYP3A4 expression (Supplementary Figure S1). Instead of *CYP3A4* genotyping, the patients’ hepatic CYP3A4 activities were estimated from CYP3A4 mRNA levels in patients’ leukocytes, categorizing the patients into low, normal and high expresser groups (Table 2). CYP3A4 expression assays revealed that more than half of the patients (56%) expressed CYP3A4 at low level, and substantial portion (43.1%) was normal CYP3A4 expressers, whereas only 2 patients (< 1%) displayed high CYP3A4 expression early after transplantation (2 days after surgery).

**Table 2:** Frequencies of *CYP3A* alleles, genotypes and CYP3A4 expression in heart transplant patients and in Caucasian population.

	N	Frequency (%)	
		Heart transplant patients	Caucasian population^a^
***CYP3A5 allele***			
*3	426	91.8	88–97
***CYP3A4 allele***			
*1B	19	4.09	3–5
*22	14	3.02	2.5–8
***CYP3A5 genotype***			
*1/*1	2	0.86	0–3.4
*1/*3	34	14.7	7.5–17.9
*3/*3	196	84.5	82–92.5
***CYP3A4 genotype***			
*1/*1	200	86.2	88–93
*1/*1B	17	7.33	6–8
*1B/*1B	1	0.43	0–4
*1/*22	14	6.03	6.6–9.2
*22/*22	–	0.0	0–0.7
***CYP3A4 expression***			
Low	130	56.0	40.2
Normal	100	43.1	47.3
High	2	0.86	12.5

^a^Allele frequencies in Caucasian population according to^38^; Genotype frequencies in Caucasian population according to^22,41,72,73^, CYP3A4 expression frequencies according to^35^.

### Patients’ CYP3A-status and tacrolimus exposure early after transplantation

**Figure 1:**
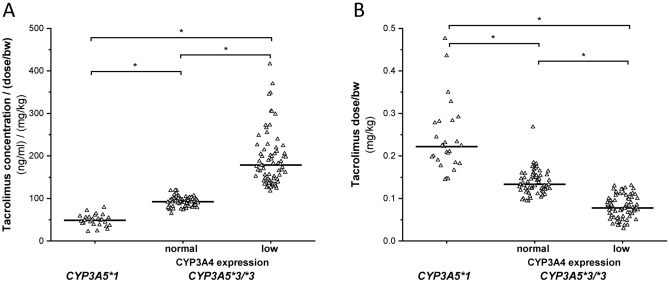
Impact of patients’ CYP3A-status (*CYP3A5* genotype and CYP3A4 expression) on the dose-corrected blood concentration and dose-requirement of tacrolimus in heart transplant recipients. (**A**) 12-h post-dose trough concentrations of tacrolimus [(ng/ml)/(mg dose/kg bodyweight)] and (**B**) dose-requirement for therapeutic blood concentration (mg/kg bodyweight) in the course of patients’ CYP3A-status are presented. The lines represent the median values of various CYP3A groups. Normal, Low: CYP3A4 expression levels. *P < 0.0001.

The association between patients’ CYP3A-status and tacrolimus blood concentration 15 days after transplantation was analysed in 163 recipients. On the basis of the post-operative CYP3A-status (*CYP3A5* genotypes and CYP3A4 expression in leukocytes), the patients were grouped into two main categories—CYP3A5 expressers (carrying CYP3A5*1/*3 or CYP3A5*1/*1 genotypes) and non-expressers (with CYP3A5*3/*3),—and the CYP3A5 non-expressers were subdivided into two subgroups: low and normal CYP3A4 expressers. Of the 163 recipients, no patient expressing CYP3A4 at high level was identified. Significant association between the recipients’ CYP3A-status and blood concentrations of tacrolimus normalized by the daily dose and the patient’s bodyweight was demonstrated (Fig. 1A). The CYP3A5 expresser patients exhibited the lowest tacrolimus blood concentrations (C_0_/D), and in CYP3A5 non-expressers, the blood concentrations were significantly higher than in those with functional CYP3A5 enzyme. Tacrolimus blood concentrations in CYP3A5 non-expressers with normal CYP3A4 mRNA level were approximately twice as high, whereas in those patients expressing CYP3A4 at low level, were about 4-times as high as in CYP3A5 expressers [CYP3A5 expressers: 48.3 ± 13.57 (ng/ml)/(mg/kg bw); normal CYP3A4 expressers: 92.6 ± 11.33 (ng/ml)/(mg/kg bw); low CYP3A4 expressers: 192.5 ± 63.60 (ng/ml)/(mg/kg bw); N = 163, P < 0.0001].

Tacrolimus dose-requirement for the therapeutic blood concentration window (10–15 ng/ml of pre-dose blood concentration) was significantly associated with the recipients’ CYP3A-status. The patients with functional CYP3A5 enzyme required higher dose of tacrolimus to reach therapeutic blood concentration than the CYP3A5 non-expressers (CYP3A5*3/*3) (CYP3A5 expressers: 0.240 ± 0.081 mg/kg bw; normal CYP3A4 expressers: 0.138 ± 0.0283 mg/kg bw; low CYP3A4 expressers: 0.080 ± 0.0266 mg/kg bw; N = 163, P < 0.0001) (Fig. 1B). Considering the basic clinical protocol for initial tacrolimus dose, the recommended dose of 0.1 mg/kg bw was not optimal for any of the CYP3A-status groups. However, modification of tacrolimus dosage adjusted to the recipients’ CYP3A-status would have been appropriate. For the recipients carrying wild-type CYP3A5*1 allele, markedly increased dose of tacrolimus (2.4-fold) was required for target blood concentration, for normal CYP3A4 expressers carrying CYP3A5*3/*3, the dose-requirement was 30–40% higher, whereas for low CYP3A4 expressers, approximately 20% lower dose was more appropriate than the recommended.

### Time-course of CYP3A4 mRNA expression and tacrolimus exposure in the first 15 months

**Figure 2:**
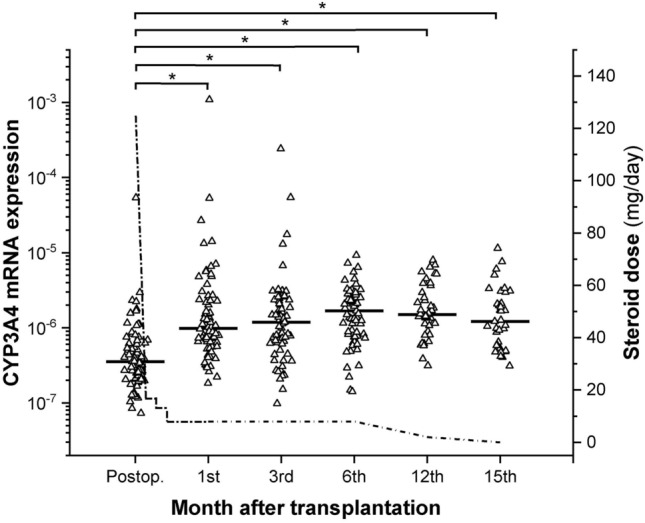
CYP3A4 mRNA expression in heart transplant recipients in a 15-month period after transplantation. The dotted line represents the daily dose of corticosteroid. *P < 0.0001.

The contribution of CYP3A-status (CYP3A4 expression and *CYP3A5* genotype) to tacrolimus exposure was investigated in 78 patients over 15 months after transplantation. In the recipients, CYP3A4 mRNA expression was significantly lower at the time of transplantation (on the second post-operative day) than at any later time points (1, 3, 6, 12 and 15 months) (P < 0.0001) (Fig. 2). At the time of transplantation, most of the patients (85%) expressed CYP3A4 at low level predicting to be poor metabolizers; however, more than three orders of magnitude difference were observed between the lowest and the highest CYP3A4 mRNA expression. One and 3 months after transplantation, although wide range of CYP3A4 expression was still observed, more than half of the recipients were intermediate metabolizers. At later time points (6 and 12 months), the CYP3A4 expression range narrowed (1–1.5 orders of magnitude), and 70–85% of the patients were intermediate metabolizers, whereas 15 months after transplantation when methylprednisolone had been withdrawn for at least one month, the proportion of intermediate metabolizers was only 47%. The resultant CYP3A4 expression during the 15-month post-transplant period was assumed to be the consequence of several non-genetic factors, e.g. corticosteroid therapy, one of the most relevant factor; therefore, methylprednisolone doses were displayed in parallel with CYP3A4 mRNA levels (Fig. 2). It should be mentioned that at the post-transplant time points of 12 and 15 months, the CYP3A4 mRNA data were incomplete due to the online control examination instead of personal consultation during COVID-19 pandemic situation.

**Figure 3:**
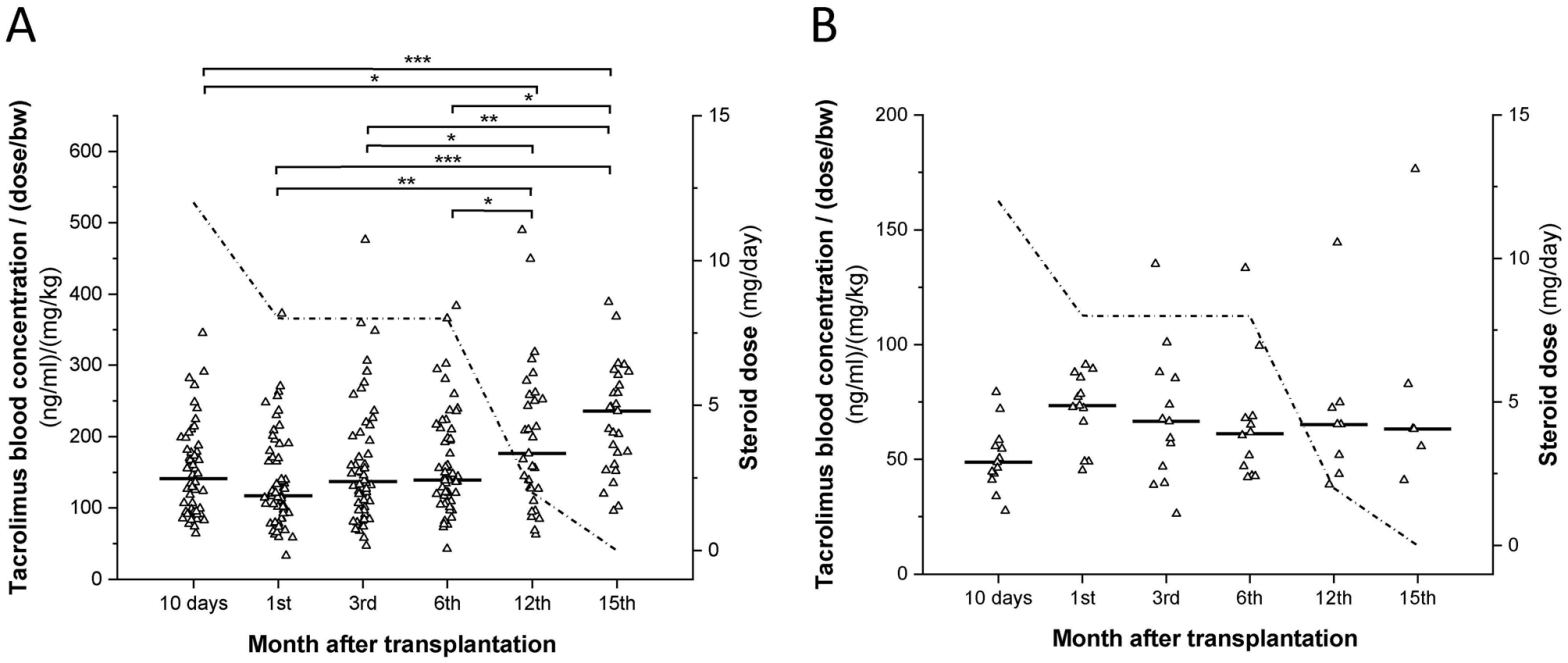
The dose-corrected tacrolimus blood concentrations in heart transplant recipients carrying CYP3A5*3/*3 genotype (**A**) and CYP3A5*1 allele (**B**) in a 15-month period after transplantation. The dotted line represents the daily dose of corticosteroid. *P < 0.05, **P < 0.001, ***P < 0.0001.

Since CYP3A5 has a dominant role in tacrolimus metabolism, fluctuation of tacrolimus C_0_ concentration per dose/bodyweight was followed separately in CYP3A5 expresser and non-expresser recipients (N = 13 and 65, respectively) (Fig. 3). Tacrolimus therapy generally started 5 days after transplantation; therefore, at the first time point, blood sampling for CYP3A4 mRNA measurement did not coincide with the sampling for tacrolimus blood concentration assay. In CYP3A5 non-expressers (with CYP3A5*3/*3 genotype), some decrease in tacrolimus blood levels normalized by the dose and the bodyweight was observed in the first month after surgery (Fig. 3A); however, it was statistically not significant (P > 0.05). Moreover, during the first 6 months, there was no significant differences in tacrolimus C_0_ concentration per dose/bodyweight (P > 0.05). On the other hand, tacrolimus blood levels 1 year or even 15 months after transplantation significantly increased comparing to those at the early time points (10 days, 1, 3 and 6 months) that were most probably due to the substantial reduction and withdrawl of methylprednisolone therapy (Fig. 3A). In CYP3A5 expressers (with CYP3A5*1/*3 genotype), no significant differences in tacrolimus blood levels per dose/bodyweight were observed between various time points (Fig. 3B). It should be noted that of the 4 concentration values higher than 125 (ng/ml)/(mg dose/kg bw), 3 values belonged to the same specific patient whose CYP3A4 mRNA expression presented extremely low levels throughout the follow-up study. As this patient displayed very low CYP3A4 expression, he/she was likely to express CYP3A5 at low level as well.

## Discussion

Considering the dominant role of CYP3A5 in tacrolimus metabolism, numerous studies have investigated the association between *CYP3A5* genotype and tacrolimus pharmacokinetics in transplant recipients. CYP3A5 expressers with at least one CYP3A5*1 allele have been reported to require higher tacrolimus doses for achieving therapeutic blood concentration than CYP3A5 non-expresser kidney transplant recipients or patients transplanted with CYP3A5 non-expresser liver grafts^34,41–43^. In CYP3A5 non-expressers (carrying CYP3A5*3/*3), the major catalyst of tacrolimus metabolism is CYP3A4. Plenty of *CYP3A4* variants have been described; however, the clinical relevance of these alleles is sometimes controversial. Amirimani et al. have demonstrated an elevated CYP3A4 transcriptional activity of CYP3A4*1B allele; however, the influence of this allelic variant on tacrolimus pharmacokinetics appears to be inconsistent most probably due to the strong genetic linkage with CYP3A5*1, the dominant enzyme in tacrolimus metabolism^44–47^. Although CYP3A4*22 allele with decreased enzyme activity appears to have an impact on tacrolimus clearance^42,48^, in the heart transplant patients in the present study, no association was found between the *CYP3A4* genotypes (for CYP3A4*1B and CYP3A4*22 alleles) and CYP3A4 mRNA expression. Due to non-genetic factors, phenoconversion might have masked the influence of genetic variants on CYP3A4 expression.

The postoperative CYP3A4 mRNA expression in heart transplant patients was significantly different from that in the healthy population. The ratio of low, normal and high CYP3A4 expressers considerably varied from the present heart transplant population to the liver donors with healthy liver function in a former study [heart transplant recipients (N = 232): 56%, 43.1%, 0.9% *vs* liver donors (N = 112): 40.2%, 47.3% 12.5%; Chi-square = 26.031; P < 0.0001]^35^. We assumed that the primary disease or the consequences of the primary disease and the surgical stress as non-genetic factors shifted CYP3A4 expression towards low CYP3A4 mRNA levels. In patients with chronic heart failure, reduction of hepatic drug clearance was attributed to a multi-component mechanism^49^. Although the exact mechanisms have not been revealed, increased hepatic venous pressure and reduced arterial oxygen saturation (hypoxia) as a consequence of weak cardiac output were assumed to induce the production of pro-inflammatory cytokines, such as IL-2 (interleukin 2), IL-4, IL-6 and IFN-γ (interferon γ). Furthermore, ischemia during surgical procedure appears to be accompanied with the release of the pro-inflammatory cytokine TNF-α (tumor necrosis factor α) and IL-6, despite the high-dose corticosteroid treatment at the time of transplantation^50^. These cytokines have been demonstrated to down-regulate the expression and function of CYP3A4 and CYP3A5 both in hepatocytes and in intestinal cells^51–56^. Furthermore, in renal transplant patients, a transient decrease in tacrolimus clearance was observed 3–4 days after transplantation that was attributed to a temporary increase in serum IL-6 concentration^56^. Although pro-inflammatory cytokine release was not determined in the present heart transplant population, it was reasonable to assume that IL-6 or TNF-α concentrations increased as a consequence of surgical interventions.

The findings of the present study confirmed a significant association between the post-operative tacrolimus blood concentration (C_0_/D) and the patients’ CYP3A-status (*CYP3A5* genotype and CYP3A4 expression). In accordance with the in vitro and clinical evidences regarding the fact that subjects carrying CYP3A5*1 allele are extensive tacrolimus-metabolizers^17,57–59^, the lowest tacrolimus concentrations normalized by the dose and the bodyweight were observed in the heart transplant patients with at least one CYP3A5*1 allele and consequently expressing functional CYP3A5 enzyme. Of the CYP3A5 non-expressers, the normalized blood concentrations in those patients expressing CYP3A4 at normal level were approximately twofold higher, and in low CYP3A4 expressers were nearly fourfold higher than in the CYP3A5 expresser patients. The relatively low standard deviations both in the CYP3A5 expresser group and in normal CYP3A4 expresser group indicated that in the patients of each group, the tacrolimus clearance was similar. However, the variation of tacrolimus clearance in the patients expressing CYP3A4 at low level was high. It means that the optimization of the initial tacrolimus therapy in patients with low CYP3A4 expression was a great challenge for clinicians. At the early post-operative time, the average daily dose required for the initial target tacrolimus concentration of 10–15 ng/ml was markedly higher for the CYP3A5 expresser patients than for the non-expressers; furthermore, significant differences in dose-requirement were observed between low and normal CYP3A4 expressers carrying CYP3A5*3/*3 genotype. According to the classical clinical protocol, the initial tacrolimus dose is suggested to be as high as 0.1 mg/kg bodyweight^4,10^. However, for the recipients with various CYP3A-statuses, the tacrolimus dosing for therapeutic blood concentrations required some modification. A substantial increase (approximately 2.4-fold) of the initial dose was necessary for the CYP3A5 expressers with CYP3A5*1 allele, whereas minor modification was required for low and normal CYP3A4 expressers carrying CYP3A5*3/*3 (20% decrease and 40% increase, respectively). Our results regarding *CYP3A5* genotype dependent dose-requirement were in line with the findings in heart transplant recipients in other studies that a substantial increase in tacrolimus daily dose was necessary for the patients carrying CYP3A5*1 allele^57,58,60,61^. In pediatric heart transplant recipients^58^, the dose-requirement of CYP3A5 expressers was similar to that in the adult recipients in the present study (0.278 and 0.240 mg/kg bodyweight, respectively)^58^. In addition to *CYP3A5* genotype, the reduced-function CYP3A4*22 allele as an inheritable factor was proposed to be integrated in pharmacogenetic prediction for personalized tacrolimus therapy^27^. However, in heart transplant recipients, CYP3A4*22 either alone or in combination with *CYP3A5* genotype was considered to provide no additional information beyond *CYP3A5* genotype^61,62^. However, CYP3A4 expression was not assessed in these studies, and no information about tacrolimus-metabolizing capacity was available in CYP3A5 non-expressers, the majority of patients. In a former study involving liver transplant patients, the donors’ CYP3A-status (*CYP3A5* genotype and CYP3A4 expression) was demonstrated to be associated with tacrolimus clearance in recipients^34^. The tacrolimus dosing however had to be modified merely in those recipients transplanted with liver grafts carrying the functional CYP3A5*1 allele and with grafts from low or high CYP3A4 expresser donors (approximately 60% of the liver transplant patients). For those with normal CYP3A4 expresser grafts, the recommended daily tacrolimus dose of 0.1 mg/kg bodyweight was appropriate for therapeutic blood concentration. In contrast, modification of the initial dose was necessary to some extent for all heart transplant patients in the present study.

Several phenoconverting factors (primary disease, surgical intervention, medication) can significantly modulate the expression of *CYP3A* genes and tacrolimus-metabolizing activity of patients in the early and late post-operative period after transplantation. In the longitudinal follow-up study, marked increase in CYP3A4 expression was observed in the first month after cardiac transplantation. The relatively low CYP3A4 expression at the time of transplantation was attributed to the primary pathological condition and to the surgical stress, and was ameliorated partly as a consequence of corticosteroid treatment. The high starting dose of methylprednisolone (as high as 250–125 mg/day) and the daily dose tapering to 8 mg by the end of the 3rd week were assumed to induce CYP3A4 transcription. Continuous increasing trend in CYP3A4 expression was observed up to 6 months that was maintained by the end of the first year when corticosteroid therapy was generally getting withdrawn. As a consequence of corticosteroid cessation, CYP3A4 expression tended to decrease. In parallel, the maximal tacrolimus-metabolizing capacity was achieved by the first month, whereas corticosteroid withdrawal abated tacrolimus clearance in CYP3A5 non-expresser patients. In those patients carrying functional CYP3A5*1 allele, tapering and cessation of corticosteroid dose however did not alter tacrolimus-metabolizing capacity. Pharmacokinetic interaction between corticosteroids and tacrolimus has been reported in renal transplant recipients, and induction of CYP3A enzymes and the efflux transporter P-glycoprotein was assumed to be the most likely mechanism of the interaction^63^. As a consequence of corticosteroid tapering and withdrawal, a significant increase in dose-corrected tacrolimus exposure has been demonstrated in CYP3A5 non-expresser kidney transplant patients^64–66^. However, no or negligible elevation in tacrolimus concentration (C_0_/D) was observed in CYP3A5*1 carriers that is consistent with the findings of the present study with heart transplant patients. Methylprednisolone and prednisolone have been reported to transcriptionally induce CYP3A4, but not CYP3A5 expression^67^ that confirmed the differences in corticosteroid dependent tacrolimus elimination between CYP3A5 expressers and non-expressers.

Some limitations of the present study should be considered. First, we did not assess *CYP3A5* and *CYP3A4* alleles other than CYP3A5*3, CYP3A4*1B and CYP3A4*22; however, the prevalence of other clinically relevant *CYP3A* alleles in Caucasian populations are extremely low^68^. Second, CYP3A4 mRNA expression clearly indicates the impact of drugs that can modify the transcription of *CYP3A4* gene; however, the patients’ CYP3A-status does not inform about the interaction with CYP3A4 inhibitors, such as fluconazole or itraconazole. These drugs can significantly decrease CYP3A4 function and tacrolimus clearance, but do not modify CYP3A4 mRNA expression. Third, beyond steroid therapy, some confounding factors, such as age and hematocrit were not taken into account during the interpretation of the results. CYP3A5 expression and activity are constant from early childhood through adulthood, whereas CYP3A4 expression exceeds the adult level by the age of 3 years, decreases to the adult level by puberty and is markedly reduced in elderly patients^69–71^. The heart transplant recipients were all above 18 at the time of transplantation, and of 232, there were only 8 patients older than 65; therefore, the patients’ age might have been considered as a minor or negligible phenoconverting factor. Tacrolimus displays strong binding to red blood cells; therefore, the alteration of haematocrit in the early post-operative period can influence tacrolimus clearance^69^. Although some decrease in dose corrected tacrolimus concentration was recognized during the first month after transplantation, it was attributed to an increase in CYP3A4 expression rather than to any alteration in haematocrit.

In conclusion, the present study involving heart transplant recipients demonstrated a significant association of the patients’ CYP3A-status (*CYP3A5* genotype and CYP3A4 expression) with tacrolimus blood concentration normalized by the dose and the bodyweight (C_0_/D) as well as with dose-requirement for optimal therapeutic blood level in the early post-operative period. Modification of the initial tacrolimus dose (0.1 mg/kg bodyweight) was necessary for all recipients. The recipients’ CYP3A-status potentially identified the degree of tacrolimus dose modification for avoiding over- or underexposure in the early postoperative period: CYP3A5*1 carriers required a 2.4-fold increase in tacrolimus dose, whereas dose reduction by 20% was appropriate for low CYP3A4 expressers, and a 40% increase in tacrolimus dose was required for normal expressers. Due to the initial high dose and later on the tapering and cessation of corticosteroid, marked increase in CYP3A4 expression by the first month, continuous elevation throughout the first 6 months and some decrease after steroid withdrawal as well as fluctuation of tacrolimus-metabolizing capacity were observed in CYP3A5 non-expresser patients, but not in those carrying CYP3A5*1. Although monitoring of tacrolimus blood concentration cannot be substituted by assaying recipients’ CYP3A-status, it can guide optimization of the initial tacrolimus dose and can also facilitate personalized tacrolimus therapy during steroid withdrawal in the late post-operative period.

## Supplementary Information

Supplementary Information.

## Abbreviations

ABCB1: ATP-binding cassette family B1
CYP: Cytochrome P450
GAPDH: Glyceraldehyde 3-phosphate dehydrogenase
IFN-γ: Interferon γ
IL: Interleukin
PCR: Polymerase chain reaction
SNP: Single nucleotide polymorphism
TNF-α: Tumor necrosis factor α
