# Supplementary material for: CYP3A-status is associated with blood concentration and dose-requirement of tacrolimus in heart transplant recipients
Source: Sci Rep. 2021 Nov 1;11:21389. doi: 10.1038/s41598-021-00942-y (PMC8560807; doi:10.1038/s41598-021-00942-y)
Supplement: Supplementary file 1 — Supplementary Information. [file 41598_2021_942_MOESM1_ESM.pdf]

Supplementary Table 1. Sequences for *CYP3A* genotyping and CYP3A4 mRNA quantification.

***CYP3A* genotyping**	**Primer/Probe**	**Sequence 5'→3'**
*CYP3A5*3*	Forward	GAG AGT GGC ATA GGA GAT ACC
	Reverse	TGT ACG ACA CAC AGC AAC C
	Wild	FAM- TTT GTC TTT CA**A** TAT CTC TTC CCT GT -BHQ1
	Mutant	CalRed610- TTT GTC TTT CA**G** TAT CTC TTC CCT GT -BHQ2
*CYP3A4*1B*	Forward	TCT GTA GGT GTG GCT TGT TG
	Reverse	AAG GGT TCT GGG TTC TTA TCA G
	Wild	HEX- TCG CCT CTC TC**T** TGC CCT TGT –BHQ2
	Mutant	FAM- TCG CCT CTC TC**C** TGC CCT TG –BHQ1
*CYP3A4*22*	Forward	CAG AGG TAG GTC TAA TTC AGT TCA
	Reverse	AGA TCA CCT TCT ATC ACA CTC C
	Wild	FAM- ATC ACA CCC AG**C** GTA GGG C -BHQ1
	Mutant	HEX- ATC ACA CCC AG**T** GTA GGG CC –BHQ2
**mRNA quantitation**		
CYP3A4	Forward	CCC ACA CCT CTG CCT T
	Reverse	GAT CAT GTC AGG ATC TGT GAT
	Probe	HEX- AGT ATG GAA AAG TGT GGG GCT T –BHQ2
GAPDH	Forward	AGC CAC ATC GCT CAG ACA C
	Reverse	GCC CAA TAC GAC CAA ATC C
	Probe	FAM-TGG GGA AGG TGA AGG TCG-BHQ1

FAM, CalRed610, HEX fluorescent labeling, BHQ1 and BHQ2 black hole quenchers

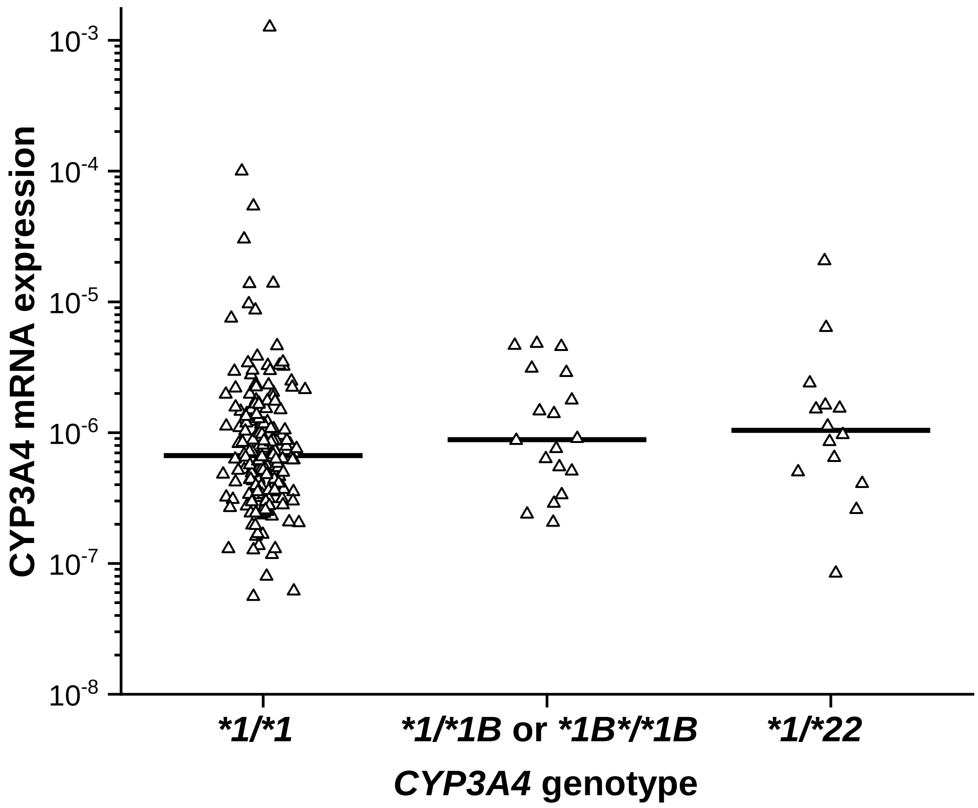

Supplementary Figure 1. Impact of *CYP3A4* genotype on CYP3A4 expression in heart transplant recipients.
